# Unusual Bone Metastasis Neuroblastoma in Adolescent With Unidentified Primary Site

**DOI:** 10.1002/ccr3.70446

**Published:** 2025-05-06

**Authors:** S. Naim, S. Sraidi, S. Salam, S. Cherkaoui, M. Lamchahab, M. Qachouh, M. Rachid, A. Madani, N. Khoubila

**Affiliations:** ^1^ Department of Hematology and Pediatric Oncology 20 August 1953 Hospital, CHU IBN ROCHD Casablanca Morocco; ^2^ Department of Pediatric Radiology Abderrahim Harouchi Hospital, CHU IBN ROCHD Casablanca Morocco

**Keywords:** bone metastasis, neuroblastoma, pediatrics and adolescent medicine oncology

## Abstract

We present a rare case of bone metastatic Neuroblastoma without a diagnosis of an identifiable primary lesion in a 16‐year‐old patient who presented with intermittent right knee pain and soft tissue swelling for 7 months. Initial radiologic examinations revealed a primary bone tumor in the distal metaphyseal‐diaphyseal region of the femur, and a follow‐up CT of the chest, abdomen, and/or pelvis revealed no evidence of a solid mass. The first bone biopsy provided inconclusive results, leading to a misdiagnosed osteosarcoma. The revisiting from St. Jude children's research hospital concluded metastasis from a Neuroblastoma. The patient was subsequently classified into the high‐risk group and given standard treatment.

## Graphical abstract

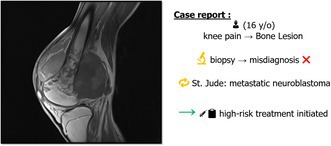

**Summary**

- In this clinical case, we emphasize a rare and atypical presentation of neuroblastoma, reported in only a few studies, highlighting the importance of considering it in practice to avoid misdiagnosis and improve patient outcomes.

## 1 Introduction

Neuroblastoma (NB) is the most common extracranial solid tumor in children. The median age at diagnosis is 19 months. It is characterized by clinical heterogeneity. Depending on the risk group, treatment ranges from observation to aggressive multimodal treatment. Stage and age are clinically predictive of outcome. Younger patients (usually defined as 18 months or younger) do better [1]. Imaging is used to assess primary tumors and determine patterns of disease spread. Nearly 60% of tumors originate in the abdomen, with 32% of cases in the adrenal glands; other sites are the chest (15%), pelvis (5%), neck (5%), and rarely the brain (1%) [2]. The Bone and bone marrow are the most frequent sites of metastatic disease, affecting skeletal structures from the skull and spine to the appendicular skeleton, followed by the liver and skin [3].

We present a rare case of a patient with bone metastasis from neuroblastoma originating from an unidentified primary site.

## 2 Case History/Examination

A 16‐year‐old adolescent presented with intermittent right knee pain and soft tissue swelling around the knee prior to admission since January 2019, 7 months before his first consultation. There was no reported family history of early‐onset cancers on either the maternal or paternal sides. A CT scan of the right knee, performed at another hospital, revealed significant osteolysis in the femur's distal metaphyseal‐diaphyseal region with early extension to the epiphyseal region and extensive soft tissue invasion. A bone biopsy was conducted, and the histological diagnosis suggested a sarcoma. However, tumor cells were immunohistochemically negative for CD99, CD3, CD20, Td, CCD45, Desmin, Myogenin, EMA, and Cytokeratin. The patient was subsequently referred to our Pediatric Hemato‐Oncology Medical Center.

## 3 Methods

**FIGURE 1:**
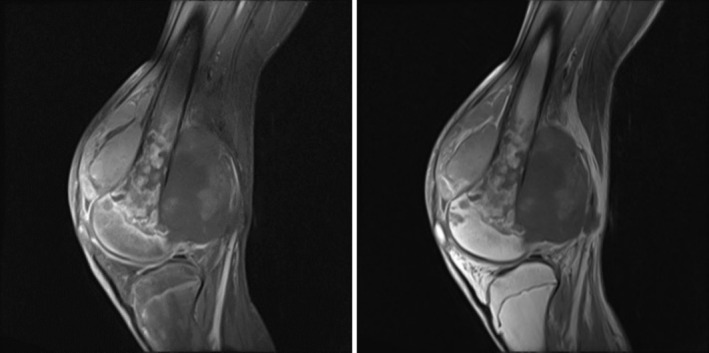
MRI of the knee showing a tumor process of the inferior femoral metaphysis enhanced after injection of gadolinium with foci of necrosis and extension to the soft parts.

The initial physical examination revealed a healthy patient, with right knee pain and diffuse soft tissue swelling around this site, accompanied by surgical scarring. The knee circumference was 42 cm, and there was no evidence of lymphadenopathy or abdominal mass. Radiographic images revealed an erosive bone lesion involving the metaphyseal‐lower diaphyseal femoral joint, as well as a soft tissue mass (Figure 1). The image was not significantly different from previous findings and was highly suggestive of a primary malignancy bone tumor, with osteosarcoma being the most appropriate diagnosis. Hematological and liver function tests were normal. Initially, no bone marrow biopsy was performed. Chest CT, abdominal CT, and pelvic CT were performed without significant abnormalities. A 99mTc bone scan showed increased tracer uptake only at the right knee. The I123 MIBG scan was canceled due to a lack of products during the covid pandemic border closure.

Pathologists from our institute reviewed the case; the bone biopsy was updated, and the diagnosis of an undifferentiated sarcoma was made on the basis of histologic and immune histochemical features. We sent it to a foreign institute for further studies in terms of cytogenetics and molecular biology. Given the location of the tumor, the patient was diagnosed with osteosarcoma and underwent three cycles of chemotherapy. The tumor process was significantly reduced radiologically and clinically, with a knee circumference of 36 cm.

Simultaneously, the results received from the St Jude institute department of pathology revealed a malignant small round cell tumor with significant crush artifact infiltrating bone. The most intact tumor fragments had a high proliferation index, with numerous mitotic and karyorrhectic cells. The anatomic location of the tumor suggests Ewing sarcoma family of tumors; however, immune histochemical results showed that the tumor cells were positive for Synaptophysin (strong) and CD56, whereas they were negative for CD99, BCOR, Cytokeratin AE1/AE3, ALK, Desmin, NB84, and Vimentin, which was also negative in the majority of the tissue. Regardless of sample limitations or unusual anatomic location, it would be important to investigate the possibility of metastasis from a neuroblastic tumor in light of the immunohistochemical finding. Unfortunately, the tissue was insufficient for further molecular genetics research.

A second bone biopsy revealed a fibrous change with no viable tumor proliferation or necrotic foci. At the time, a bone marrow biopsy revealed no abnormalities.

Our medical center's multidisciplinary consultation determined that the patient was diagnosed with metastatic neuroblastoma with an unknown primary site after extensive imaging, including a CT scan, bone scan, and the I123 MIBG scan, which revealed no primary site, and the patient received standard treatment as the high‐risk group neuroblastoma.

## 4 Conclusion and Results

This report outlines the clinical characteristics of a patient with neuroblastoma metastasis in the bone without an apparent primary site. The challenge was to determine the origin of this neoplasm. Our patient's imaging studies, which included CT scans, revealed no evidence of a primary tumor. Another distinguishing feature of this case is the radiological appearance of the bone lesion, which strongly suggests the presence of a primary bone tumor such as osteosarcoma. This case could easily be misdiagnosed as Ewing's sarcoma or osteosarcoma without a thorough examination of the pathological specimens. Other information, such as histology, MYC status, or marrow involvement, was not available for our patient due to a variety of factors, including the following: insufficient tissue for additional molecular genetic studies, the second bone biopsy revealed a fibrous alteration without viable tumor proliferation or necrotic foci, and the absence of a molecular biology bench in our anatomopathology laboratory.

**FIGURE 2:**
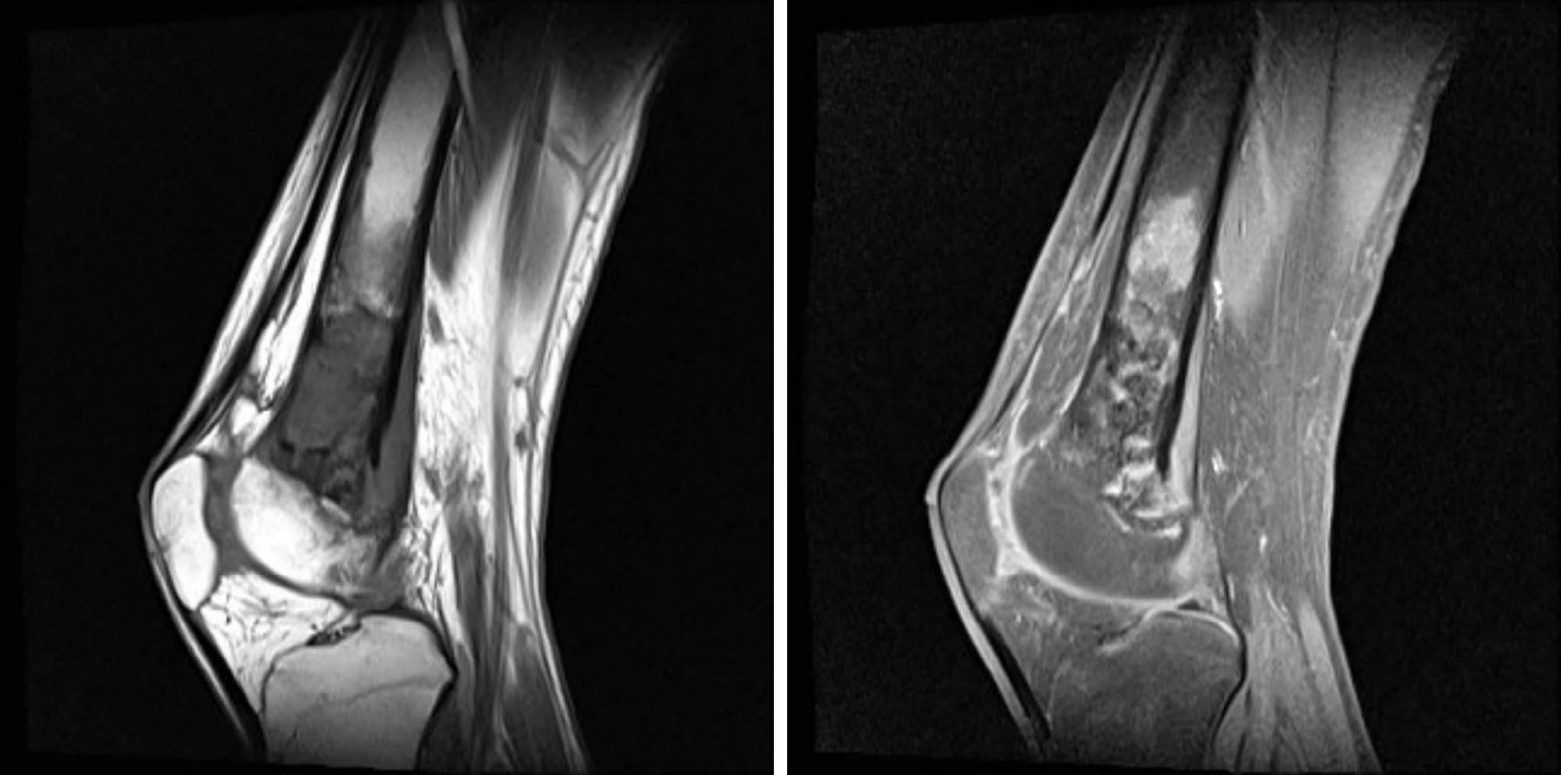
Control MRI after chemotherapy showing a clear reduction in the volume of the tumor process.

The patient received five cycles of induction chemotherapy. Preoperative MIBG 1131 scintigraphy showed no pathological detection in the entire examination volume, especially the right femur (no viable residual tumor) prior to surgical resection. Fibrous and hemorrhagic changes with some foci of necrosis, four cycles of consolidation therapy followed by maintenance therapy with 13‐cis‐retinoic acid. Sixty months after the end of treatment, the patient is still in complete remission (Figure 2).

Despite extensive investigation, we conclude this is a rare case of Neuroblastoma without a known primary site. Given that only a few cases of neuroblastomas with unknown primary sites are reported to date, it is difficult to determine whether they have special features that differentiate them from other metastatic neuroblastomas. However, this unique type of NB should be better understood and researched in order to improve treatment and prognosis.

## 5 Discussion

Neuroblastoma is the second most common solid tumor in children after brain tumors. It accounts for 8%–10% of all malignant tumors in children and originates in neuroblasts of the adrenal medulla and sympathetic nervous system. 70% of NBs develop metastases, and prognosis is closely related to age at diagnosis, clinical disease, and biological factors. The International Neuroblastoma Risk Group (INRG) classification system was developed to create risk stratification based on patient and tumor characteristics prior to treatment. High‐risk neuroblastoma accounts for half of all neuroblastomas and is associated with < 50% long‐term survival [4].

Clinically, patients with neuroblastoma present with palpable abdominal abnormalities and/or complaints related to mass effect on adjacent organ systems, such as extremity edema, shortness of breath, and bone pain. Metastasis is frequently encountered upon initial presentation (60%–70%), and the extent of metastatic lesions is correlated with prognosis and staging [5]; Neuroblastomas originating from extra‐abdominal sites might be associated with more favorable clinical and biological characteristics and a better outcome than neuroblastomas originating from the abdomen [6].

After a review of the literature, fifteen additional cases of patients with similar features were found. Clinical and histopathologic features, imaging studies performed, and oncologic treatment were either incomplete or absent in the majority of these reported cases. Over an 11‐year period, only four cases (5%) of 79 patients with high‐risk neuroblastoma were diagnosed at a single institution [7]. Only the characteristics and outcomes of four of these patients were reported. All four patients were in stage IV and had bone metastasis, three of them in the skull and the fourth in an unspecified location. Three patients received chemotherapy and radiation, while the fourth received chemotherapy only. One had no evidence of recurrence at the end of the study periods, one had local recurrence, and two had died [7, 8, 9, 10] (Table 1).

**TABLE 1:** Patient characteristics of neuroblastoma in the bone or bone marrow without an apparent primary site (7, 8, 12, 13).

Authors	Age	Sex	Diagnosis	Metastasis	Treatment	Outcame
Our case report (2019)	16 year old	Boy	Neuroblastoma	Right knee bone	Induction Monobloc resection Consolidation	72 months; No Evidence of disease (NED)
Nino Rainusso et al. (2019)	23‐month‐old	Boy	Undifferentiatd Neuroblastoma	Right eye orbit	Induction, consolidation: Radiotherapy: 24Gy right orbit, AHSCTx1 Maintenance: Cis—retinoicacid (6 cycles)	120 months; NED
Nino Rainusso et al. (2019)	7‐year‐old	Girl	Undifferentiatd Neuroblastoma	Lower extremities, pelvis, and vertebrae	Induction, Consolidation: AHSCT×1, Maintenance: Cis—retinoicacid (6 cycles) Immunotherapy	96 months; NED hearing loss
Nino Rainusso et al. (2019)	11‐year‐old	Boy	Undifferentiatd Neuroblastoma	Left distal femur	Induction, consolidation: Radiotherapy: 24Gy left distal femur, AHSCT×1 Maintenance: Cis‐retinoicacid (6 cycles)	69 months; NED
Nino Rainusso et al. (2019)	7‐year‐old	Girl	Poorly Differentiated Neuroblastoma	Lower extremities, pelvis, and vertebrae	Induction Consolidation: AHSCT×2 Maintenance: 6 cycles Cis—retinoicacid	144 months; NED; premature ovarian failure
Wei Zhang et al. (2016) [11]	3‐year‐old	Boy	Neuroblastoma	Osseous lesions Mandibular and sphenoid bones	Chemotherapy toward neuroblastoma was initiated	Residual neuroblastoma
Darren Salmi et al. (2010)	20‐month old	Girl	Neuroblastoma	Periorbital Mass Bone marrow	DFCI 34‐DAT Protocol local radiation, and autologous hematopoietic stem cell transplantation	over 4 months post‐AHSCT, disease‐free
Conte (2006) [12]	16 years old	Girl	Neuroblastoma	Bone and bone marrow	Chemotherapy (no information)	Dead of disease

Abbreviations: AHSCT×1, single autologous hematopoietic stem cell transplant after high‐dose chemotherapy with etoposide, carboplatin, and melphalan; AHSCT×2, tandem autologous hematopoietic stem cell transplant after high‐dose chemotherapy with etoposide, carboplatin, cyclophosphamide, and melphalan and total body irradiation (12Gy).

Until now, 1% of patients had no detectable solid mass tumor, and there was no clear reference guideline for the treatment of these types of NB. Patients were primarily treated with chemotherapy according to the guidelines for stage IV NB treatment, because of the two sites involved and the lack of a detectable primary tumor [13].

## Author Contributions

**S. Naim:** conceptualization, methodology, resources, validation, visualization, writing – original draft, writing – review and editing. **S. Sraidi:** supervision, visualization. **S. Salam:** resources. **S. Cherkaoui:** supervision. **M. Lamchahab:** supervision. **M. Qachouh:** supervision. **M. Rachid:** supervision. **A. Madani:** supervision. **N. Khoubila:** conceptualization, methodology, resources, supervision, validation.

## Ethics Statement

For the case report, we obtained patient consent for publication with medical record confidentiality.

## Consent

We obtained written informed consent.

## Conflicts of Interest

The authors declare no conflicts of interest.

## Data Availability

The data that support the findings of this study are available from the corresponding author upon reasonable request.
